# Supplementary material for: Complexome profiling of the Chlamydomonas psb28 mutant reveals TEF5 as an early PSII assembly factor
Source: Plant Cell. 2025 Mar 17;37(6):koaf055. doi: 10.1093/plcell/koaf055 (PMC12164590; doi:10.1093/plcell/koaf055)
Supplement: koaf055_Supplementary_Data [file koaf055_supplementary_data.zip › psb28_interactive.zip!/plots/intensity/qualityPlots/peptides/ALB3.1_Cre06.g251900.t1.2.html]

### Cre06.g251900.t1.2

Trivial name: ALB3.1  
SeqCoverage [median]: 16.10  
Mass: 43.41 kDa  
  
 

| Sequence | MissedCleavage | Mass [Da] | wtIntensity | muIntensity | Score |
| --- | --- | --- | --- | --- | --- |
| AEAEEAVER | 0 | 1002.46 | 3786.13 | 460.67 | 124.23 |
| ARAEAEEAVER | 1 | 1229.60 | 6475.36 | 2949.01 | 93.62 |
| LGGANVVMNELGPVTKPGSGR | 1 | 2052.08 | 2541.49 | 4326.42 | 100.67 |
| LTSLVQDGSTASAAVAGASA | 0 | 1775.89 | 6183.68 | 1190.31 | 100.02 |
| QQVESAMAVQALKPR | 1 | 1654.88 | 4066.05 | 2170.48 | 127.27 |
|  |  |  | 23052.7 | 11096.9 |  |
